# A Case of Pericardial Effusion and Human Immunodeficiency Virus in the Postmodern Era

**DOI:** 10.7759/cureus.33349

**Published:** 2023-01-04

**Authors:** Rosaida Silverio Lopez, Pankaj Agrawal

**Affiliations:** 1 Internal Medicine, South Georgia Medical Center, Valdosta, USA; 2 Hospital Medicine, South Georgia Medical Center, Valdosta, USA

**Keywords:** antiretroviral therapy, tuberculosis, viral pericarditis, hiv, pericardial effusion

## Abstract

Pericardial effusion is a relatively common cardiac pathology associated with various infectious and non-infectious etiologies. In developed countries, viral pericarditis and idiopathic reasons are the two most common causes of this condition. Mycobacterium tuberculosis is prevalent in developing countries and is the most common cause of pericardial effusion in these regions. Parasitic and bacterial etiologies are encountered less frequently. In this report, we describe the case of a large pericardial effusion in a patient with HIV and latent tuberculosis (TB). Pericardiocentesis and analysis of pericardial fluid did not reveal any specific etiology, indicating viral or idiopathic pericarditis as an etiology. We also present an analysis of global data related to pericardial effusion in HIV/AIDS patients, and the impact that the increasing availability of antiretroviral therapy (ART) worldwide over the last three decades had had on it. The CD4 count has been described as an essential factor for the prognosis of this condition. Patients with lower CD4 count levels would be at higher risk of severe pericardial effusion.

## Introduction

Pericardial effusion refers to the accumulation of fluid between the visceral and parietal layers of the pericardium that exceeds the standard pericardial fluid collection, which, in a healthy individual, is about 15-50 ml and is serous in nature [1]. Etiologies of pericardial effusion and its prevalence vary geographically, and it reflects the variations in access to treatment in different regions. Over the last three decades, a significant decrease in the incidence and prevalence of HIV-related pericardial effusion has been observed in developed countries with the emergence of antiretroviral therapy (ART). However, the change has been more gradual in developing countries. Similarly, with regard to tuberculosis (TB), there is mounting evidence that TB patients in the HIV era are likely to have disseminated illness, and have a pericardial disease that is culture-positive, with a high incidence of short- and long-term poor outcomes. Tuberculous pericarditis is the most common manifestation of TB heart disease, with a 40% death rate in 2019 among ART-naïve HIV patients. However, there is scarce historical data to assess the incidence shift with regard to anti-tuberculous treatment and pericardial effusion. Recent studies have shown poor penetration of TB drugs into the pericardium [2], which could explain why constrictive pericarditis, one of the most severe complications of TB pericarditis, affects between 17 and 60% of patients despite them receiving the necessary anti-tubercular treatment [3]. We report a unique case of a patient with two significant risk factors for pericardial effusion, HIV, and latent, treated TB-associated pericardial effusion due to viral or idiopathic pericarditis.

## Case presentation

**Figure 1:**
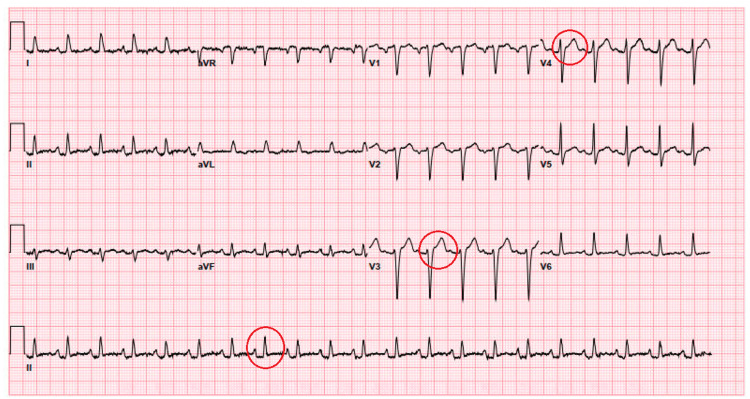
EKG obtained upon arrival showing multiple ST-segment elevations (red circles) EKG: electrocardiogram

**Figure 2:**
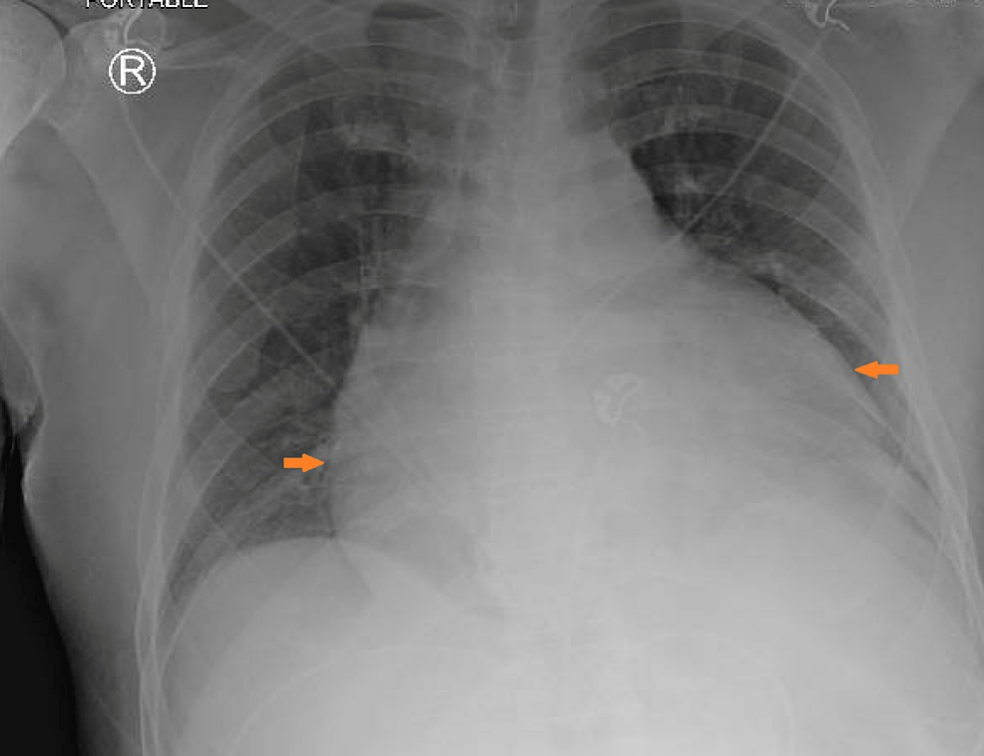
Chest X-ray at the time of initial presentation demonstrating cardiomegaly (arrows)

The patient was a 62-year-old African American male, with a past medical history of HIV, latent TB, chronic kidney disease stage 3a, essential hypertension, hyperlipidemia, and type 2 diabetes mellitus, who was brought to the emergency department by ambulance with complaints of a one-day history of chest pain, body aches, and shortness of breath. His symptoms worsened with a cough and had no apparent alleviating factors. On arrival, his vital signs showed a temperature of 101.1 °F and a heart rate of 129 beats per minute. His blood pressure was 132/65 mmHg, and his respiratory rate was 19, with an oxygen saturation of 95% on room air. He did not show any signs of acute distress on physical examination. A cardiac examination demonstrated tachycardia. He had been on elvitegravir, cobicistat, emtricitabine, and tenofovir alafenamide (Genvoya) for HIV. He had not been aware of his CD4 count before arrival. He had no previous reported history of opportunistic infection. He conveyed that he had completed treatment for his latent TB in the distant past. On arrival, EKG showed ST elevation in multiple leads (Figure 1), but his troponin was within normal limits. Other initial parameters were as follows - WBC: 7.4 x 10^9^/L, hemoglobin: 11.0 g/dL, platelet count: 266 10^3^/µl, erythrocyte sedimentation rate (ESR): 70 mm/hr, C-reactive protein (CRP): 9 mg/dL, and lactic acid: 0.70 mmol/L. As for imaging studies, cardiomegaly was present on his chest X-ray (Figure 2), and an echocardiogram showed a moderate to large pericardial effusion. A previous echocardiogram about 18 months ago had been within the normal range. The patient was hemodynamically stable with no signs of cardiac tamponade, but a pericardiocentesis was deemed necessary due to the volume of fluid observed on the echocardiogram.

Approximately 700 mL of serosanguinous fluid was extracted through a subxiphoid pericardiocentesis the next day after his presentation. The pericardial fluid analysis was indefinite, showing only blood and proteinaceous debris and rare, intact, and benign-appearing mesothelial cells. No atypical cytologic features were seen. The acid-fast smear for Mycobacterium was negative. Further serum testing showed that his antinuclear antibody was in the normal range and his CD4 count was 265 cells/mm^3^. A Karius test was performed and revealed the presence of Epstein-Barr virus (EBV). However, an antibody panel later demonstrated that this was not an acute EBV infection. The patient received empiric antibiotics and continued improving symptomatically, but the definitive cause of the effusion remained undetermined. Since this was a large effusion without a sign of tamponade, it was likely a subacute or chronic effusion with acute viral or idiopathic pericarditis. Medication-induced pericardial effusion has been described, specifically with isoniazid. Our patient reported undergoing treatment for latent TB in the remote past, and hence this was an unlikely cause.

## Discussion

A pericardial effusion is defined as an excessive accumulation of fluid around the heart between two layers of the pericardium. It usually amounts to 15-50 ml in quantity and is serous in nature in a healthy individual [1]. Pericardial effusion is classified based on the time of onset into acute, subacute, or chronic (>3 months); based on size found on echocardiogram imaging into mild (<10 mm), moderate (10-20 mm), or large (>20 mm); based on location into circumferential or loculated; and based on composition into transudate or exudate [4].

Etiologies

Common etiologies of pericardial effusion can vary from infectious and inflammatory to rheumatologic, neoplastic, vascular, cardiac, and idiopathic [1]. The frequency of each etiology varies widely and has changed over time; thus, there is a lack of universal consensus on it. A recent study of 269 consecutive patients who underwent pericardiocentesis at a university hospital between 2006 and 2016 showed that 26% of cases were idiopathic, 25% malignancy-related, 20% were iatrogenic, and 7.4% were related to infections. Both viral and bacterial infections were noted to have a similar frequency, which included one case of HIV (0.4%) [5]. However, a study published in 1993 involving 53 patients at the Veterans’ Administration Medical Center showed malignancy (23%), viral infection (14%), idiopathic (14%), collagen vascular disease (12%), and uremia (12%) as the leading causes of large pericardial effusion [6]. Pericarditis is seen as an underlying mechanism behind pericardial effusion in a large number of these etiologies, which could be acute, subacute, or recurrent. Acute pericarditis is found in about 5% of patients visiting the ED and 0.1-0.2% of hospitalized patients with non-ischemic chest pain [7]. Causes of acute pericarditis include but are not limited to infection (viral, bacterial, Mycobacterium), autoimmune, trauma, chest trauma, cancers, and toxins [8]. TB continues to be the major cause of pericarditis in developing countries, whereas it accounts for less than 5% of cases in developed countries. Viral or idiopathic pericarditis remains the most common cause of pericarditis in developed countries, representing about 80-90% of cases [9].

Pathophysiology

Pericardial effusion refers to an excess amount of fluid collection in the pericardial sac, which could be exudative, transudative, or serosanguinous. In acute conditions, only 100-150 ml of fluid can lead to pericardial tamponade due to the low elasticity of the pericardium. An increasing amount of pericardial fluid affects cardiac hemodynamics in multiple ways. It begins by compressing the right side of the heart due to its thinner wall, which leads to reduced diastolic filling causing venous congestion. This diastolic filling reduction also affects the left ventricle and limits the stroke volume, causing low cardiac output and hypotension. In a chronic setting, pericardial fluid can accumulate up to 2 liters in quantity before the signs of tamponade manifest [1].

Clinical presentation

Most patients with a pericardial effusion show no symptoms specific to pericardial effusion unless they reach a point of tamponade. Patients may have symptoms of underlying causes such as chest pain, fever, and malaise in cases of acute pericarditis. The clinical symptoms in a patient with a large pericardial effusion include but are not limited to chest pain, generalized weakness, chest discomfort in a supine position, and shortness of breath. Most diagnoses are made during the evaluation of other medical conditions with an echocardiogram, chest X-ray, and CT scan of the chest.

Diagnosis

Clinical assessment, EKG, and chest radiograph help to determine the presence of pericardial effusion, but echocardiography is ultimately needed for an accurate diagnosis. If there is a non-diagnostic echocardiogram but a strong suspicion of pericardial effusion, cross-sectional imaging with CT or MRI is advised. Creatine kinase-MB (CK-MB) and troponin are two cardiac biomarkers that may be elevated in pericardial effusion. The first steps after its discovery involve assessing a pericardial effusion's size, hemodynamic significance, particularly the presence of cardiac tamponade, and potentially related disorders. In up to 60% of instances, pericardial effusion is accompanied by a known disease, and the treatment depends on the underlying medical condition [4].

Pericardiocentesis plays an essential role in the patient’s symptomatic improvement and the pericardial fluid analysis to identify the effusion's specific etiology (Table 1) [10]. However, the results can sometimes be inconclusive, leading to challenges in the treatment approach. Light’s criteria for pleural effusion are commonly used to assess pericardial effusion due to the similarity of the components being analyzed. Recently published data have described that the composition of pericardial fluid is distinct and that comparing its content to pleural fluid could lead to diagnostic errors [11]. Table 2 presents a comparison highlighting important differences. The pericardial fluid is rich in protein, albumin, lactate dehydrogenase (LDH), and nucleated cells with low levels of glucose and cholesterol. The culture of the fluid for the growth of bacteria and acid-fast smear for Mycobacterium are also critical when considering causes such as TB.

**Table 1:** Main analyses performed on pericardial fluid and suggested etiologies LDH: lactate dehydrogenase; CEA: carcinoembryonic antigen; CYFRA: cytokeratin; TB: tuberculosis; PCR: polymerase chain reaction Obtained with permission from Zdravniški Vestnik [10]. Published on October 31, 2020

Analysis	Test	Etiology
Biochemical tests	Specific weight >1.015. Proteins >30 g/L, punctate/serum ratio >0.5. LDH >2 mCat/L, punctate/serum ratio >0.6. Glucose, leukocytes	Exudate
Cytologic tests	Neoplastic cells	Neoplasm
Biological markers	CEA >5 ng/L or CYFRA 21-1 >100 ng/mL. Adenosine deaminase >40 U/L, interferon-gamma	Neoplasm
PCR	TB-PCR	TB
Microbiological tests	Bacilli staining. Aerobic and anaerobic cultures	TB, other bacteria

**Table 2:** Comparison of normal reference intervals of pericardial fluid to transudative pleural fluid *Value is two-thirds of the upper limit of normal for those aged 18 years or older (222 IU/L). ^†^Similar to blood glucose concentration LDH: lactate dehydrogenase Obtained with permission from Heart [11]. Publisher: BMJ. Published on September 14, 2021

	Pericardial fluid	Pleural fluid transudate
Biochemical components		
Total protein (g/dL)	2.8 (1.7-4.6)	<3.0
Total protein ratio	0.5 (0.29-0.83)	0.5
Albumin gradient (g/dL)	1.4 (0.18-2.37)	1.2
LDH (U/L)	357 (141-2613)	147*
LDH ratio	1.1 (0.40-2.99)	0.6
Glucose (mg/dL)	95 (80-134)	65-139^†^
Total cholesterol (mg/dL)	27 (12-69)	45
Cellular counts		
Leucocytes (10^6^ cells/L)	503 (35-2210)	125 (83-214)
Mesothelial cells (10^6^ cells/L)	1283 (40-3790)	1 (0-2)
Lymphocytes (10^6^ cells/L)	304 (19-1634)	23 (16-31)
Polymorphonucleated cells (10^6^ cells/L)	2 (0-116)	1 (0-2)
Macrophages (10^6^ cells/L)	1 (0-207)	75 (64-81)

In 2006, a study by Mayosi et al. [12] analyzed the results of pericardiocentesis in 69 patients with pericardial effusion and its correlation with HIV (Table 3). They compared the results of chemistry studies like adenosine deaminase (ADA), and microscopic analysis, with Ziehl-Nielsen stain for acid-fast bacilli testing, and Mycobacterium tuberculosis cultures. They found no significant differences in clinically HIV-infected and those who were not regarded as having a high ADA level or the presence of acid-fast bacilli on microscopic analysis.

**Table 3:** Results of pericardial fluid analyses by clinical HIV status HIV: human immunodeficiency virus; TB: tuberculosis Obtained with permission from BMC Infectious Diseases [12]. Publisher: Springer Nature. Published on January 6, 2006

Feature	Clinical HIV disease, n (%)	No clinical HIV disease, n (%)	P-value
Pericardiocentesis indication	31 (44.9)	38 (55.1)	0.29
Diagnostic	14 (45.2)	17 (54.8)	0.97
Therapeutic	17 (44.7)	21 (55.3)	
Pericardial aspirate analyses			
Adenosine deaminase			
>40 IU/L	8 (42.1)	11 (57.9)	0.79
<40 IU/L	7 (46.7)	8 (53.3)	
Ziehl-Nielsen stain for acid-fast bacilli			
Positive	3 (42.9)	4 (57.1)	0.62
Negative	20 (40.8)	29 (59.2)	
TB culture			
Positive	2 (33.3)	4 (66.7)	0.61
Negative	4 (40.0)	6 (60.0)	

Treatment

Patients who have a small, asymptomatic pericardial effusion without tamponade should be monitored but will not typically require prompt intervention. Patients who are symptomatic and have a significant effusion need to undergo pericardiocentesis and receive additional care. The medical emergency of cardiopulmonary tamponade necessitates immediate drainage. To avoid the pericardial effusion getting worse or returning, the underlying cause must be found and treated.

**Figure 3:**
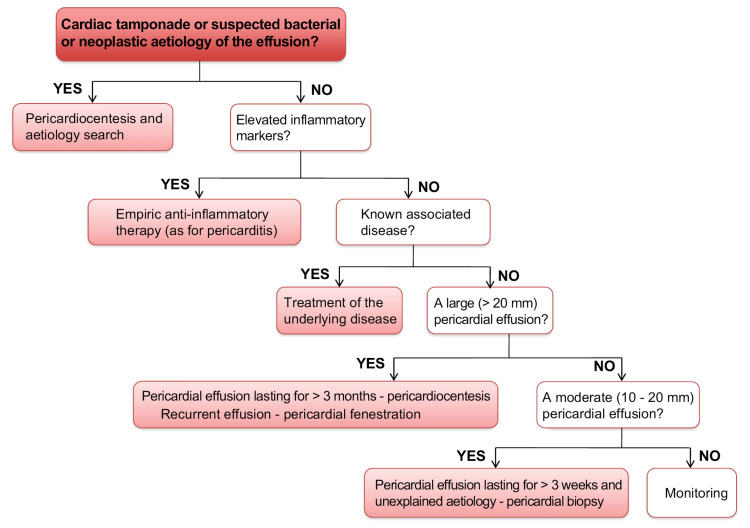
Triage and management algorithm of pericardial effusion Reviewed by the Council of the Slovenian Medical Association on September 8, 2020, in agreement with the European Society of Cardiology guidelines of 2015 Obtained with permission from Zdravniški Vestnik [10]. Published on October 31, 2020

In tubercular pericardial effusion, anti-tubercular therapy is recommended. Intravenous antibiotic treatment is used to treat purulent pericardial effusion. Both Gram-positive and Gram-negative bacterial infections should be treated with an empirical antibiotic regimen. Vancomycin, third-generation cephalosporins like ceftriaxone, and carbapenems including imipenem are the most commonly used antibiotics. It is advised to maintain intravenous antibiotic therapy for two to four weeks until all clinical indications of infection have disappeared. Non-steroidal anti-inflammatory drugs (NSAIDs) and supportive treatment are employed to treat viral pericarditis that results in pericardial effusion. Pericardiocentesis or surgical drainage are both options for treating cardiac tamponade. Both procedures are highly effective in removing fluid and relieving symptoms related to hemodynamic compromise. Catheter pericardiocentesis is described by the 2015 European Society of Cardiology guidelines as the preferred treatment for most patients and the drainage catheter should be kept until the daily drainage is less than 25 mL (Figure 3) [10].

Prognosis

The prognosis is dependent on the cause and severity of the effusion. Idiopathic effusion usually has a good prognosis when the size is mild to moderate. Large idiopathic chronic effusions have a 30-35% risk of progression to cardiac tamponade [4].

Pericardial effusion in HIV/AIDS: historical progression and the global shift with use of antiretroviral therapy

Heidenreich et al. published a five-year prospective study, conducted between 1988 and 1993, which demonstrated the high prevalence and incidence of pericardial effusion in AIDS patients. This study also estimated that the incidence of pericardial effusion was 11% per year in asymptomatic AIDS patients, and the presence of pericardial effusion was associated with a poor clinical outcome (36% survival at six months) [13]. Patients with a lower CD4 count were noted to be at higher risk of pericardial effusion [14]. Zidovudine (AZT), a nucleoside reverse transcriptase inhibitor (NRTI), was the first antiretroviral medication demonstrated to have beneficial effects on clinical development and mortality in 1987. Multiple resistance mutations emerged because of sequential monotherapy. The mid-1990s introduction of protease inhibitors (PIs) and non-nucleoside reverse transcriptase inhibitors (NNRTIs) revolutionized the treatment of HIV infection. The combination regimens consisting of two NRTIs plus a PI or NNRTI caused virological suppression and its use showed a reduction in morbidity and mortality [15]. ART became widely available to patients with HIV/AIDS in 1996 [16]. The prognosis of pericardial effusion in HIV/AIDS patients has been positively influenced by ART.

Himelman et al. [17], in the year 1989, published a study in which 70 HIV-positive patients were evaluated for cardiac manifestations of HIV. They found pericardial effusion in seven (10%) hospitalized patients. Among the seven patients, six were hospitalized for the treatment of active Pneumocystis carinii pneumonia. One had a previous history of the infection, two had a history of Kaposi’s sarcoma, one had pulmonary TB, and one had non-Hodgkin’s lymphoma; dilated cardiomyopathy was also seen in three of the patients. This demonstrated that the patients had multiple comorbidities and advanced disease when pericardial effusions were evidenced. Zidovudine was the only therapy available at the time of the study, although it was not described if they were receiving treatment or not. Heidenreich et al., in 1995 [13], reported a 36% survival at six months in patients with AIDS who had pericardial effusion versus 93% survival at six months in AIDS patients without pericardial effusion. This was one of the first studies to describe mortality related to AIDS and pericardial effusion. This study did not mention if the participants were receiving ART. In 2011, Lind et al. studied the incidence of pericardial effusion in the antiretroviral era. They reported that only two of 802 (0.25%) HIV-infected patients showed pericardial effusion in echocardiography and none of those patients exhibited tamponade or signs of cardiovascular impairment such as swinging heart [18].

The Joint United Nations Programme on HIV/AIDS (UNAIDS) data show that in 2005, about 63% (24.5 million) of the world’s HIV population lived in Sub-Saharan Africa. In 2021, this came down to about 50% (25 million). Of these, only 30,000 HIV patients had access to ART in the year 2002, which increased 25 folds by the year 2005 to 800,000, and in 2021, 80% (20 million) of them had access to ART. In 2021, about 82% of HIV infected population in Western and Central Europe and North America reportedly had access to ART [19,20]. In 2003, Magula et al. published an extensive review of the literature published from January 1980 to February 2003 on cardiac disease in HIV patients living in Africa. The most common HIV-related cardiac abnormalities were cardiomyopathy and pericardial disease. TB was the primary cause of large pericardial effusion in Africa. About half of hospitalized patients and a significant proportion of patients followed up over several years developed cardiac abnormalities [21]. The World Health Organization (WHO) reported that between 2000 and 2021, new HIV infections fell by 49%, and HIV-related deaths fell by 61% with 18.6 million lives saved thanks to ART in the same period. The WHO African Region is the most affected region, with approximately 25.6 million people living with HIV in 2021. Also, the WHO African Region accounts for almost 60% of the new global HIV infections, which is still comparable to values in 2008. It would be of utmost importance to continue implementing strategies for ART expansion and disease complication control, including for cardiovascular disease and opportunistic infections [22].

## Conclusions

The overall incidence of HIV-related pericardial effusion has decreased drastically with the use of ART globally. The availability of ART in developing countries has been on the rise over the last two decades. It is imperative that global leaders work to maintain and expand access to ART for HIV-positive patients as this guarantees fewer health complications including cardiovascular disease. It is also important to consider that although patients on ART rarely develop pericardial effusion, they are still susceptible and should be evaluated when symptoms and risk factors indicate this condition.
